# Joint Evolution of Kin Recognition and Cooperation in Spatially Structured Rhizobium Populations

**DOI:** 10.1371/journal.pone.0095141

**Published:** 2014-04-24

**Authors:** Peter C. Zee, James D. Bever

**Affiliations:** Department of Biology, Indiana University, Bloomington, Indiana, United States of America; Universidad Carlos III de Madrid, Spain

## Abstract

In the face of costs, cooperative interactions maintained over evolutionary time present a central question in biology. What forces maintain this cooperation? Two potential ways to explain this problem are spatially structured environments (kin selection) and kin-recognition (directed benefits). In a two-locus population genetic model, we investigated the relative roles of spatial structure and kin recognition in the maintenance of cooperation among rhizobia within the rhizobia-legume mutualism. In the case where the cooperative and kin recognition loci are independently inherited, spatial structure alone maintains cooperation, while kin recognition decreases the equilibrium frequency of cooperators. In the case of co-inheritance, spatial structure remains a stronger force, but kin recognition can transiently increase the frequency of cooperators. Our results suggest that spatial structure can be a dominant force in maintaining cooperation in rhizobium populations, providing a mechanism for maintaining the mutualistic nodulation trait. Further, our model generates unique and testable predictions that could be evaluated empirically within the legume-rhizobium mutualism.

## Introduction

The evolution and maintenance of cooperative traits in nature is a central question in evolutionary biology. Hamilton's rule [1] states that for an altruistic trait to evolve, the costs to the actor must be outweighed by the benefits to recipients. However, this benefit must be weighted by their genetic relatedness (*C<rB*). Two ways that Hamilton's rule can be satisfied are if the population is structured [2], [3] or if the actors can recognize and direct beneficial behaviours to genetically similar individuals [4], [5].

Population structure may play a major role in the evolution of cooperation. In a population structured by local dispersal (e.g., in a viscous environment), neighbouring individuals are more likely to share a common ancestry than in a fully mixed environment. This viscosity allows the kin of a cooperative individual to receive more benefit than unrelated individuals. Conversely, in an unstructured environment, the benefits of the cooperative behaviour are equally likely to affect the fitness of any genotype. Experimental studies have shown that cooperative traits are lost during extended evolution in unstructured environments [6] and favoured in a structured environment [7].

Kin recognition allows organisms to discriminate behaviour towards kin and may enhance the likelihood of cooperative traits evolving. If individuals can preferentially direct cooperative behaviours toward kin, unrelated individuals will not receive the benefit, resulting in lower fitness. The role of kin recognition in nature is unclear and debated in the literature [8]–[10]. These kin recognition mechanisms often entail a cost of expression [11].

Here, we present a model to investigate the joint effects of spatial structure and kin recognition on the evolution of cooperation, allowing us to disentangle the relative contribution of each. To ground our study in a biological system, we modelled intraspecific cooperation among rhizobia in the biological context of the interspecific plant-microbe mutualism. Within this mutualism, there is an important component of intraspecific cooperation within the bacterial population, as the rewards of nitrogen fixation are potentially available to many bacterial individuals [12]–[14]. Rhizobia are an ideal biological system for our study because: *(i)* the rhizosphere is a spatially structured environment [15]; *(ii)* rhizobia have a greenbeard-like recognition mechanism (rhizopines) [16]; *(iii)* the nodule environment locally increases the carrying capacity of rhizobium populations, assuaging concerns regarding the strength of local competition [17], [18].

## Biological scenario: *the rhizobium – legume mutualism*

Rhizobia are soil bacteria that engage in a mutualistic interaction with leguminous plants, for which they can fix nitrogen otherwise unavailable to the plant. In this resource exchange mutualism, the bacteria receive carbon from the host plant. Rhizobia cells infect plant root cells, where they differentiate into bacteroids inside a tumor-like growth on the root called a nodule. In these nodules, bacteroids fix atmospheric nitrogen in exchange for carbon, but also stimulate the plant to release nutrient rich resources into the surrounding rhizosphere [19], [20].

Rhizobia carry a locus encoding the ability to nodulate plant roots on extrachromosomal, symbiotic plasmids [21]. Cells with functional alleles (*Nod+*) will infect plant roots, differentiate into bacteroids, and fix nitrogen. These are mutualists. Nodulation not only offers the plant benefit, but also offers an indirect intraspecific benefit to other rhizobia because it increases local resources which benefit both free-living cells in close proximity to the nodule and undifferentiated cells within the nodule (henceforth called ‘*adjacent*’). Hence, *Nod+* bacteria are also cooperators. We recognize two potential costs of functional *Nod+*genes: energetic costs of plasmid carriage and sterilization (as differentiation can be terminal) or growth rate inhibition due to differentiation into bacteroids. In this work, we focus on the energetic carriage cost of the plasmid (See Text S1 for a brief discussion on costs of nodulation). As *Nod−* bacteria receive the exuded benefits of nodulation, but do not pay the costs, they disproportionally benefit from nodulation (i.e., “cheaters”).

We follow the approach of Bever and Simms [14], and assume a fixed *cost of plasmid carriage*, *c_N_*. The basal fitness of the *Nod−* cells is set to unity, while the basal fitness of mutualistic cells is (1−*c_N_*) in all environments. The fitness of rhizobia cells also depends on their surrounding environment. Population densities of soil microbes are highest in the immediate vicinity of plant roots, where exuded nutrients are concentrated in the soil [19], [20]. Nodulation stimulates a local increase in these exudates from roots, allowing for increased bacterial population densities around nodules [17], [18]. This increase in resources available to cells adjacent to nodules is the *benefit of nodulation*, *b_N_*, to all rhizobia. The fitness of undifferentiated cells adjacent to nodules is thus increased by a factor of (1+*b_N_*); around un-nodulated roots, fitness is 1 (i.e., the standing, background availability of resources in the soil). As local population density increases, concerns have been raised regarding the potential ability of kin competition to offset the benefits of spatial structure [22]. However, these concerns are misplaced given that carrying capacity locally increases within and around nodules [23], [24]. This biological system presents a situation with elastic local carrying capacities, similar to that shown for opines in *Agrobacterium tumefaciens* [18].

In addition to the mutualistic *Nod+* locus, rhizobia can also carry loci for the production and catabolism of rhizopines (*Rhiz*) [25], [26]. Rhizopines are carbon-rich compounds produced by the plant after stimulation by nodulating bacteroids [26], [27]. Like the benefits of nodulation, rhizopines are available as nutrients to rhizobia within and in close proximity to the nodule. However, non-rhizopine individuals (i.e., *Rhiz−* individuals) are unable to catabolize rhizopines [16], rendering rhizopines a private resource for *Rhiz+* individuals [27]. Effectively, this constitutes a kin recognition system equivalent to a “greenbeard” trait [28]–[30], where the production of rhizopines is the “greenbeard”, and the unique ability for rhizopine catabolism ensures directed benefits. Like *Nod*, the *Rhiz* loci are also carried on an extrachromosomal plasmid, and production is coupled with nodulation [26]. When a *Rhiz*+ cell generates a nodule, we assume a *proportion, d, of general root exudates are diverted* to rhizopine production, reducing the resources available to *Rhiz*− cells. In addition, the synthesis and catabolism of rhizopines involve a carbon cost, *c*, that detracts from the total exudate available to all bacteria. We note that rhizopines have the characteristics of a “spiteful” trait [31] because it is costly for both the cells expressing the trait (*c*) and the non-rhizopine individuals (*d*). As with the *Nod* locus, we assume an energetic carriage cost of the *Rhiz+* allele, *c_r_*. *Rhiz+* cells occur in the population at frequency equal to *e*, while *Rhiz−* have frequency equal to *f ( = 1−e)*.

**Figure 1:**
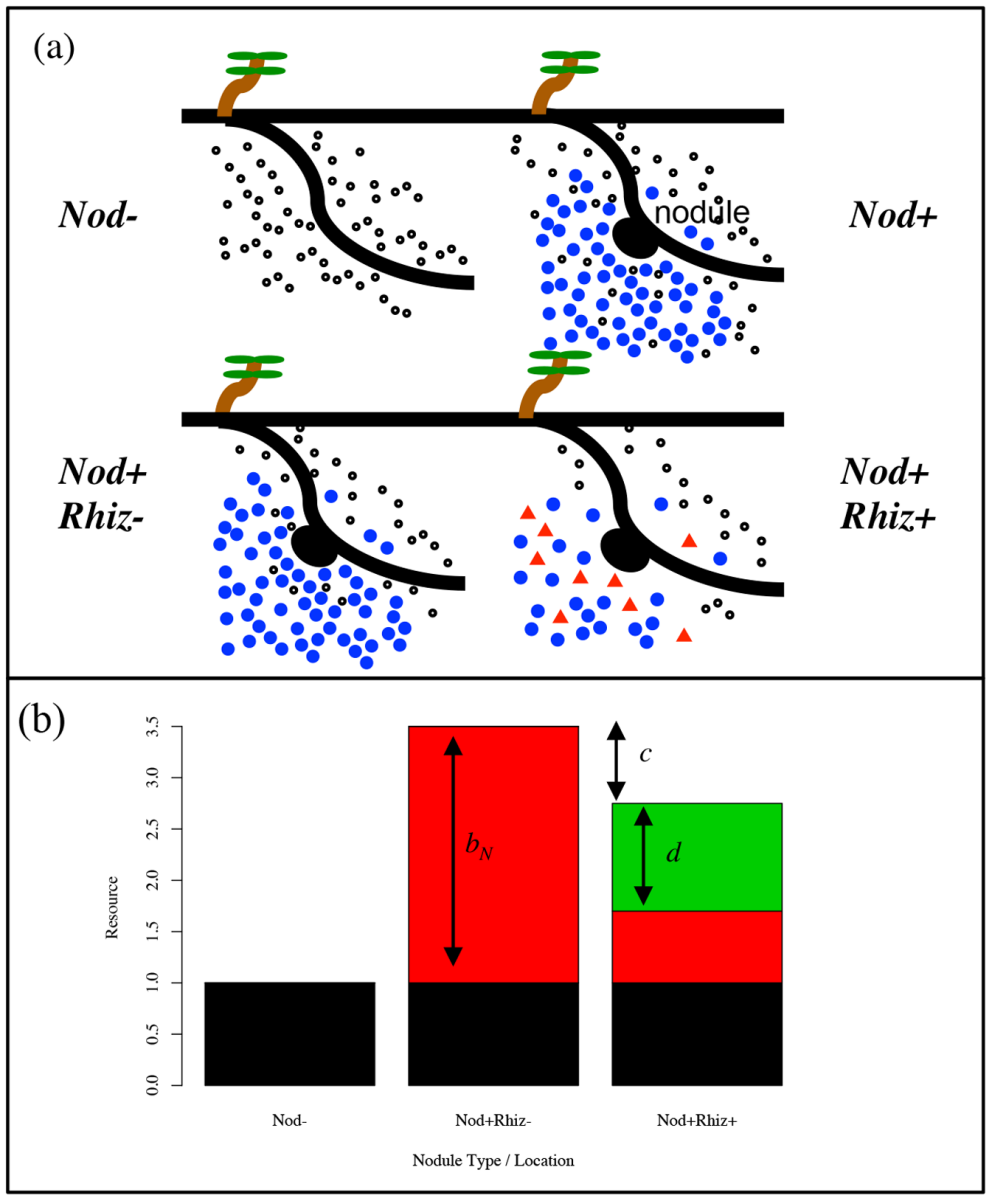
Root exudates and local resource environments. (a) Schematic of root exudates in the model. Small open circles are *general exudates* that are usable by any free-living cells. Blue circles are *nodulation induced* exudates (*b_N_*), also available to all free-living cells. Red triangles are *rhizopines*, which are only available to *Rhiz+* cells. (b) Resources in local environments. Black portions of the bars represent the *general* exudates that are usable by all types. Red portions of bars show general use exudates induced by nodulation. Green portions of the bar represent the *rhizopines*. In the *Nod+Rhiz+* bar, the two costs of *Rhiz* (*c* and *d*) can be seen to decrease the induced benefits of nodulation.

Access to increased exudates from nodulation will depend on the structure of the soil environment. The probability of a nodule forming on a plant root at any given time is dependent on the genotypic constitution of the rhizobium population present at the infection site. In an environment with no spatial structure (i.e., complete mixing), every genotype of reproductive rhizobia is equally likely to be adjacent (either within or in close proximity) to the nodule, and thereby equally likely to receive the benefits of nodulation. Conversely, in a spatially structured environment (i.e., limited mixing), reproductive cells receiving benefits of nodulation will be more likely to be of the same genotype as the nodule-founding bacteroid. We describe the level of environmental mixing with a *coefficient of relationship*, *φ*, between the bacteroid generating a nodule and the rhizobia adjacent to the nodule. This coefficient of relationship can take on values between zero and one, where *φ* = 0 represents the situation where there is complete mixing (i.e., no spatial structure), and *φ* = 1 represents a completely viscous environment (i.e., complete spatial structure). When *φ* = 1, the benefits of nodulation go exclusively to *Nod+* cells; when *φ* = 0 these benefits are randomly distributed with respect to genotype. Model parameters are summarized in Table 1. Figure 1 displays the distribution of exuded resources in the soil (a), and graphical annotations of costs and benefits (b).

**Table 1:** Description of model parameters.

Parameter	Biological meaning	Model
*x*	*Nod+* frequency	unlinked
*y* ( = 1−*x*)	*Nod−* frequency	unlinked
*e*	*Rhiz+* frequency	unlinked
*f* ( = 1−*e*)	*Rhiz−* frequency	unlinked
*x*	*Nod+Rhiz+* frequency	linked
*y*	*Nod+Rhiz−* frequency	linked
*q*	*Nod−Rhiz+* frequency	linked
*z*	*Nod−Rhiz−* frequency	linked
*φ*	Spatial structure; probability that bacteroids are identical to vegetative cells exterior of nodule	both
*b_N_*	Benefit of nodulation to surrounding cells	both
*c_N_*	Cost of carrying *Nod+* allele	both
*c_r_*	Cost of carrying *Rhiz+* allele	both
*c*	Cost of rhizopine production/synthesis; decreased general exudate output	both
*d*	Amount of exudate produced not usable by *Rhiz−* (i.e., rhizopines)	both

Spatial structure (*φ*) has opposing influences on the evolution of nodulation and rhizopines in rhizobium populations. Bever and Simms [14] showed that in sufficiently structured environments, the legume-rhizobia interspecific mutualism can be maintained through the intraspecific cooperation. However, when mixing in the population became too high, *Nod−* cells are increasingly likely to receive the benefits of nodulation, and the magnitude of the relative benefit to *Nod+* does not outweigh costs of being a mutualist. Negative frequency-dependent dynamics at the *Nod* locus result in a stable internal equilibrium, and introducing spatial structure alters the location of this equilibrium, with increasing spatial structure shifting it towards fixation of *Nod+*. Conversely, Simms and Bever [32] found that the evolution of rhizopines is facilitated in well-mixed populations fixed for *Nod+* (i.e., in a mutualistic population). When the environment is well mixed, the advantage of kin recognition (i.e., rhizopines) is high because it allows for private sharing of resources among *Rhiz+* cells. However, when the environment is highly spatially structured, local groups of cells are likely to be related, thereby eroding the advantage of the directed benefits of rhizopines, and magnifying the cost. This is because increasing spatial structure raises the likelihood of a cell being adjacent to kin, which makes paying a cost to direct rhizopines to kin superfluous because benefits are likely to reach kin with such a mechanism. This translates to positive frequency-dependent dynamics at the *Rhiz* locus, with an unstable internal equilibrium. Increasing spatial structure decreases the equilibrium frequency of *Rhiz+*, thus widening the initial conditions that lead to loss of rhizopines.

## Model

We analyze a population genetic model with two di-allelic loci (one for nodulation, *Nod*, and one for rhizopine, *Rhiz*) that are either inherited independently (no linkage; *Unlinked case*) or coinherited (complete linkage; *Linked case*). Prior research has identified spatial structure as being a key determinant of the dynamics at these loci. In this work, we focus on whether the kin recognition system of rhizopines qualitatively alters the evolutionary fate of the mutualism. We focus on the spatial structure term *φ* because we are primarily interested in how environmental structure influences the evolutionary dynamics in the rhizobium population, and its role in the legume-rhizobia mutualism.

## Model I: Unlinked case

### Independent inheritance of *Nod* and *Rhiz*

We analyze an unlinked model as a heuristic to understand the dynamics of the two loci independently. This approach assumes that dynamics of alleles of the two loci are unconstrained by linkage. While this assumption is biologically unreasonable because plasmids are not independently distributed among bacterial cells and physical linkage likely alters the transitory dynamics, analysis of the unlinked model allows application of analytical tools to capture the qualitative dynamics of the system around the equilibria. This gives us an analytical point of comparison for the more biologically realistic linked model discussed below, and in fact, the equilibria are unaffected by linkage.

We calculate fitness at the *Nod* and *Rhiz* loci as the product of two functions, *G* and *E*, that measure the constitutive growth and environment specific growth, respectively. *G* is a function of the constitutive costs and benefits (i.e., *c_N_*, *c_r_*) of a genotype. These fitness effects are experienced across all environments. *E* is a function of genotype frequency, environment-specific costs and benefits (i.e., *d*, *c*, *b_N_*), and the level of environmental mixing (*φ*). Together, these two functions measure the costs and benefits of being in each nodule environment, and the probability of being in each environment. Full exposition of the model can be found in Text S2.
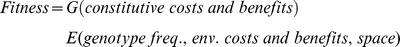
Because the loci are segregating independently, the system of equations can be reduced to two equations by noting that allele frequencies at each locus sum to unity (e.g, *x = (1−y)* (*Nod* locus) and *e = (1−f)* (*Rhiz* locus)). This reduction to two equations allows us to visualize the dynamics on a standard phase plane, with a zero net growth isocline for each locus. Changes in allele frequencies at both loci can be derived from these fitness equations (Text S2), allowing us to monitor the joint influence of spatial structure and recognition (i.e., rhizopines) on cooperation (i.e., nodulation).

## Results I: Unlinked case

**Figure 2:**
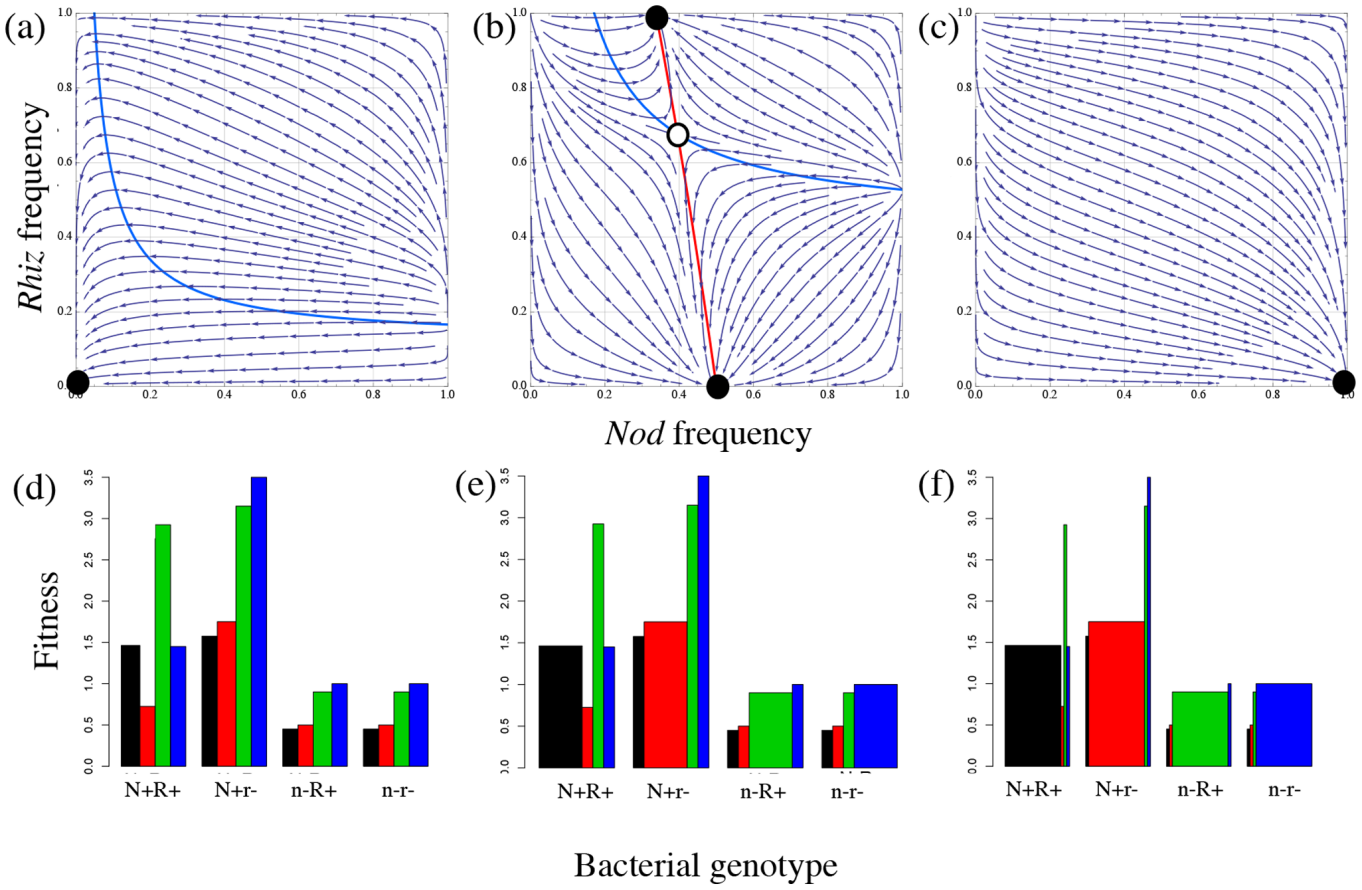
Dynamics and fitness of the unlinked model. (a–c) Isoclines and dynamics. Zero growth net growth isoclines for the unlinked model for three different levels of spatial structure (*φ* = 0, 0.5, 1). The blue, curved isocline represents the equilibrium for the *Rhiz* locus and is unstable. The linear isocline is the equilibrium for the *Nod* locus and is stable. Vectors on the phase plane represent the evolutionary dynamics towards the equilibria. (d–f) Fitness of genotypes in each nodule environment. These panels of display the fitness of each cell type in each environment (i.e., nodule adjacency). Width of bars is proportional the probability of being found in that environment, as altered by degree of spatial structure (*φ* = 0, 0.5, 1). Black, red, green and blue bars (left to right within each cluster of bars) represent *Nod+Rhiz+*, *Nod+Rhiz−*, *Nod−Rhiz+* and *Nod−Rhiz−*, respectively.

First, increasing spatial structure in the population greatly facilitates the evolution of mutualism (Figure 2). With elevated spatial structure undifferentiated *Nod+* individuals will tend to be adjacent to nodules and able to receive the full benefits of the root exudates (Figure 2f). Alternatively, as the environment becomes increasingly mixed, nodulation is less likely to evolve (Figure 2a), because the carriage cost associated with nodulation (*c_N_*) is not recovered through the indirect benefits of root exudates because *Nod−* individuals are increasingly likely to receive the benefit (Figure 2d). This retrieves the result of Bever and Simms [14] modelling the evolution of nodulation in a population without rhizopines.

Second, increasing spatial structure constrains the evolution of rhizopines (Figure 2b). In well-mixed environments, benefits of rhizopines are directed to other *Rhiz+* individuals. However, as spatial structure increases and *Rhiz+* individuals tend to be clustered, the relative benefits of kin recognition decrease because of a decreasing need for preferential allocation. Without non-rhizopine genotypes to compete with, rhizopines offer no advantage, and *Rhiz+* cells suffer the cost of rhizopine production. Additionally, some level of mixing in the environment must occur for the maintenance of the kin recognition system. These results recover those of the Simms and Bever [32] modelling the evolution of rhizopines in a population fixed for *Nod+*.

Finally, increasing frequencies of rhizopines decrease the equilibrium frequency of *Nod+* (illustrated by the negative slope of the *Nod* isocline in Figure 2b; Figure S1). The dependence of the evolution of cooperation on kin recognition can be derived as the partial derivative of the *Nod*+ frequency with respect to *Rhiz*+ frequency:
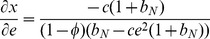
This expression is always negative as long as costs of producing rhizopines (*c*) are positive. (This expression is undefined when *φ* = 1, a biologically unreal scenario). This indicates that, at equilibrium, rhizopines (i.e., kin recognition) are not beneficial to the maintenance of cooperation when not coinherited with the *Nod* locus. This result emerges because costs of rhizopine production act as an additional cost to the cooperative *Nod* trait, which leads to a decrease in cooperation at equilibrium.

## Model II: Linked case

### Coinheritance of *Nod* and *Rhiz*

We now consider a more realistic linked model, where the *Nod* and *Rhiz* loci are coinherited on the same plasmid; this is equivalent to complete linkage between the two loci. This is more biologically realistic than the unlinked case because these loci are often located on the same symbiotic plasmid in rhizobium species [33], [26]. We now follow four distinct two-locus genotypes: *Nod+Rhiz+*, *Nod+Rhiz−*, *Nod−Rhiz+*, and *Nod−Rhiz−*. The fitnesses of these genotypes are calculated in the same way as in the unlinked case, as the product of the functions *G* and *E*, with *x*, *y*, *q*, and *z* denoting the frequencies of the four genotypes, respectively. A key distinction between the unlinked and linked models is that in the linked case, the costs and benefits of rhizopine production are coinherited with the costs and benefits of the nodulation. This makes it possible for rhizopines to more directly influence the evolution of nodulation, thus altering the transient genotype dynamics.

Unlike the unlinked, this linked model does not lend itself to analytical tractability. However, we use several approaches to understand the qualitative behaviour of the model in relation to the results from the unlinked model. First, we analyze the invasion conditions for each genotype. By assuming fixation of one genotype, we can determine which (if any) of the three remaining genotypes can invade by comparing fitnesses. While this approach allows us to qualitatively determine which genotypes are stable at fixation, it does not reveal any information regarding internal dynamics. We use numerical iterations of the full model to map genotype dynamics over time. Finally, we turn to a weak-selection approximation of the model that allows us to plot internal dynamics. From these three approaches, we are able to achieve a qualitative understanding of the linked model.

## Results II: Linked case

**Figure 3:**
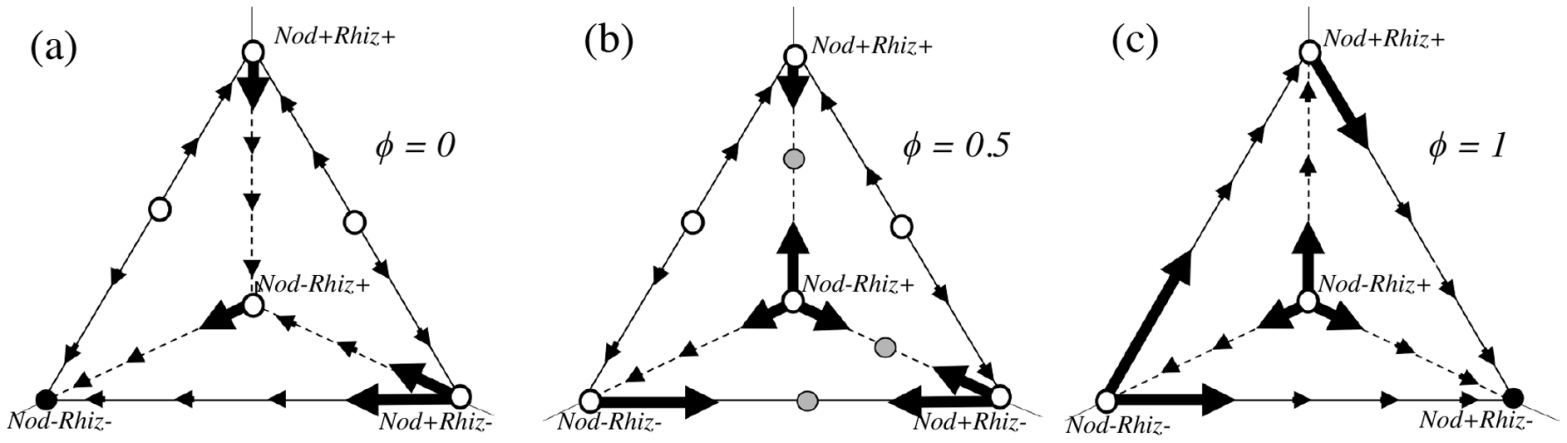
Invasibility at different levels of spatial structures. (a–c) Increasing levels of spatial structure (*φ* = 0, 0.5, 1). Black filled circles represent stable equilibria, grey filled circles represent unstable internal equilibria, while open circles are unstable. Arrows represent the movement of the population along the edges of this genotype space.

The invasibility of genotypes in the linked model changes with spatial structure (Figure 3). At low spatial structure (Figure 3a), the non-interacting genotype (*Nod−Rhiz−*) is globally stable. All other genotypes are invaded by this genotype because it is able to reap benefits of general exudates, yet suffers none of the costs. As spatial structure increases (Figure 3b), we see that no genotype is globally stable; rather, the equilibrium is a polymorphic population. This matches the results of the unlinked model, where an increase in spatial structure leads to the evolution of nodulation. When the environment is highly structured (Figure 3c), the system moves towards the *Nod+Rhiz−* genotype, as in the unlinked model. These invasion results echo the qualitative dynamics of the unlinked case, and the quantitative conditions for the stable corner equilibria (*Nod+Rhiz−* and *Nod−Rhiz−*) are identical. Invasion criteria for each of the genotypes are presented in terms of *φ* in Text S3.

At intermediate values of *φ*, the invasion analysis cannot determine the stability of internal equilibria. There are potential internal equilbria (Figure 3c; gray circles) at three locations in the genotype-space: *(i)* between the *Nod+Rhiz+* and *Nod−Rhiz+* genotypes; *(ii)* between the *Nod−Rhiz+* and *Nod+Rhiz−* genotypes; and *(iii)* between the *Nod+Rhiz−* and *Nod−Rhiz−* genotypes. To investigate the stability of these points, we turn towards numerical simulation and analytical approximations.

**Figure 4:**
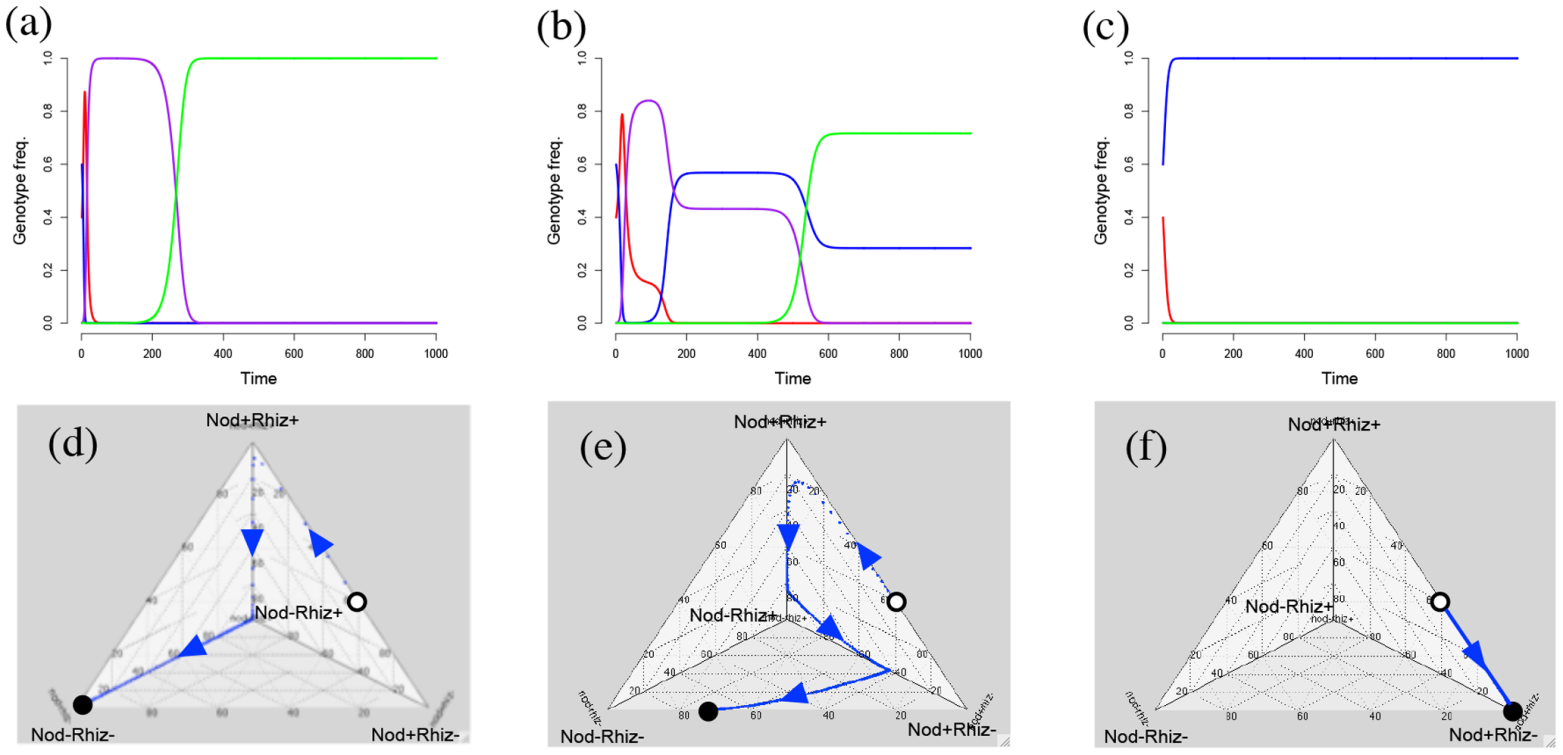
Linked genotype frequency dynamics. (a–c) Genotype frequency dynamics of the linked model for φ = {0, 0.5, 1}. *Nod+Rhiz+*, *Nod+Rhiz−*, *Nod−Rhiz+* and *Nod−Rhiz−* are represented by red, blue, purple, and green lines, respectively. At low spatial structure, there is a transient increase in *Nod+Rhiz+* frequency (red line). At higher spatial structures, this increase disappears, and *Nod+Rhiz−* goes to fixation. Genotype frequency is plotted on the y-axis, and time in generations is on the x-axis. (d–f) Evolutionary dynamics in the genotype space simplex. Blue arrows represents evolutionary trajectory, and black point represents the evolutionary endpoints. Open circles show initial condition.

Numerical simulations show that increasing the spatial structure greatly facilitates the evolution of cooperation (Figure 4). In structured environments, *Nod+Rhiz−* quickly sweeps to fixation (Figure 4c,f). In less structured populations, the *Rhiz+* allele facilitates a transient increase in the frequency of cooperation. At this intermediate level of mixing, we see that stable equilibrium is a population polymorphic for the *Nod+Rhiz−* and *Nod−Rhiz−* genotypes (Figure 4b,e), as in the unlinked model. In unstructured populations, the non-interacting, saprophytic genotype (*Nod−Rhiz−*) invariably fixes (Figure 4a,d). In Text S4, we discuss a weak selection approximation that enables model simplification. With these functions, it is possible to visualize isoplanes for each of the genotypes (Figure S2).

The transient increase seen in the frequency of the *Nod+Rhiz+* at intermediate and low spatial structure is a striking result of the linked model (Figure 4b,e). The magnitude of the transient gain in nodulation – quantified as the area under the curve of the *Nod+Rhiz+* frequency dynamics that is greater than the equilibrium frequency reached by the *Nod+Rhiz−* genotype – measures the increase in frequency of mutualism that would not be realized in the absence of rhizopines. Though this transient gain in frequency of cooperation is not sustained over evolutionary time, the magnitude of increase is highest in mixed environments (where the evolution of cooperation is otherwise restricted), but disappears as spatial structure increases (Figure S3).

The *Nod+Rhiz+* genotype is not stable; it is invaded by the *Nod−Rhiz+* genotype (Figure 4a,b,d,e) as it approaches fixation, leading to the pattern of transience. The *Nod−Rhiz+* genotype is a non-cooperative ‘cheating’ genotype. However, this genotype is in turn unstable (Figure 3). Where the population moves in genotypic space after this invasion is determined by the level of spatial structure: in more structured environments, the population will move to a stable polymorphism of *Nod+Rhiz−* and *Nod−Rhiz−* (Figure 4b,e); in mixed environments, the non-interacting genotype (*Nod−Rhiz−*) will sweep to fixation (Figure 4a,d).

## Discussion

The evolution and maintenance of cooperative traits in the face of countervailing forces is a longstanding question in population biology. Here, we have shown that spatial structure plays a dominant role relative to that of kin recognition in the evolution of cooperation. Spatial structure both promotes the evolution of cooperative traits, as well as maintains them over evolutionary time. When environments become increasingly mixed, non-cooperative individuals easily invade cooperative populations. We used the analytical results from the unlinked model as a basis for comparison to the more intractable linked model, which recovered many of the qualitative results.

We show that while kin recognition can favour the evolution of cooperation, it is a transient effect. As recognition-enabled cooperation becomes common, it becomes vulnerable to invasion by non-cooperative kin recognition ‘cheaters’. Moreover, we show that in some cases, kin recognition – because of additional costs – actually constrains the evolution of cooperation. This instability suggests that cooperation founded solely upon kin recognition mechanisms is unlikely. The evolutionary stability of kin recognition in the population is reliant on the sustained association between kin recognition and the cooperative trait. If these can be uncoupled from each other, genotypes that do not suffer the cost of expression will be able to invade the system. Additional instability of kin recognition stems from the fact that effective kin recognition requires fidelity between two elements: the production of a signal and the ability to recognize that signal [29], [30], [34]. The potential disentanglement of these elements can further destabilize cooperation reliant on kin recognition mechanisms. In rhizobia, the different genes underlying rhizopine synthesis and catabolism could independently mutate, generating destabilizing genotypes (i.e. genotypes that can catabolize rhizopines but do not bear the costs of rhizopine synthesis genes). By contrast, cooperation based on spatial structure does not share the same vulnerabilities to cheating as kin recognition. The analogous destabilization of cooperation based on spatial structure requires evolution of increased dispersal in non-cooperators [35]. However, constraints on the evolution of genotype-specific dispersal phenotypes are more severe than the simple loss of function mutation required in kin recognition systems. Bacteria, for example, can swim through the soil via flagella or cooperative swarming [36], [37], but even these local scale mobilities can be curtailed by drying of the soil [38]. As a result, spatial structure is a more resilient mechanism for increasing the frequency of contact between cooperators.

Though our study strengthens the view that population structure is the dominant factor in the evolution of cooperation, it does not exclude the possibility that kin recognition mechanisms are important in social evolution. In fact, the transient increase we find occurs in relatively unstructured environments, where cooperation is otherwise unlikely to evolve. Indeed, the presence of rhizopine genotypes in rhizobium populations [26], [39], [40] suggests an evolutionary force is maintaining rhizopines. One potential mechanism for this maintenance of polymorphism is broader meta-population dynamics, with migration among patches. The scale of a rhizobium-plant mutualism meta-population is not easy to identify. Due to overlap of root systems of individual plants in a local area, individual plants are unlikely to be the limit of a patch in a meta-population model. Rather, the root systems of a local cluster of plants may be more meaningful patches, and populations of bacterial cells could disperse among groups of plants. The transient increase in *Nod+Rhiz+* cells in populations will increase opportunity for migration of *Nod+Rhiz+* cells to other subpopulations, thereby maintaining rhizopines in nature. In this framework, the variation in local carrying capacities among groups could be explicitly incorporated. This meta-population model for the maintenance of rhizopines should be a focus of future study.

Our work does not consider an active role of the plant in the interaction with rhizobia. Rather, we model the evolutionary dynamics within rhizobia populations and treat the plant host as a static interactor through which cooperative benefits are delivered (i.e., exudates). This approach complements research seeking to understand the influence of plant ‘sanctions’ – the plants physiological ability to discontinue or restrict carbon allotment to nodules infected by relatively ineffective rhizobium strains – on the system dynamics [41]. These studies focus on the interspecific dynamics of this interaction, and specifically how the plant can alter the bacterial populations. If plants can “choose” among genotypes of rhizobia partners, this process can stabilize the mutualistic interaction [42], [43]. Alternatively, if plants ‘sanction’ or preferentially allocate resources among nodules, these interspecific forces can maintain the mutualism as well [41], [44]. Both of these interspecific processes presume that plant resources are reliably delivered to kin of nodulating rhizobia. Rather than address these interspecific processes, we have focused on the mechanisms underlying this reliable delivery of resources. As a result, our modeling approach is complementary to research efforts to understand the importance of partner choice, sanctions and preferential allocation in maintaining interspecific mutualisms.

By focusing our attention on how intraspecific rhizobia dynamics can maintain the legume-rhizobia mutualism, our work offers a unique perspective on this ecologically important interaction. The qualitative results from our model generate predictions for the changes in rhizobia genotype frequencies over time. Experimental tests of these predictions would be valuable contributions to understanding the evolution of the legume-rhizobia mutualism. By empirically evaluating these within-rhizobia predictions, we can move towards a more complete view of the ecology and evolution of the mutualism.

Our model suggests that spatial structure can be a dominant contributor to the maintenance of mutualistic genotypes in rhizobium populations relative to the directed benefits of rhizopines. Rhizopines (kin recognition) are removed from the population because they are vulnerable to invasion by non-cooperating ‘cheater’ genotypes (*Nod−Rhiz+*), which in turn are unstable. That the non-interacting genotype invariably goes to fixation in unstructured populations is indicative of the necessity of spatial structure of cooperation, and thus the maintenance of the plant-rhizobium mutualism. This disintegration of the mutualism is analogous to the experimental work where cooperative traits are lost during evolution in unstructured environments (*interspecific*: [45], [46]; *intraspecific*: [6]).

## Supporting Information

Figure S1 **Equilibrium frequency of nodulation is limited by rhizopines.** The equilibrium *Nod+* frequency is shown as function of spatial structure. The solid curve shows the equilibrium level of cooperation in the absence of rhizopines, while the dashed curve represents the equilibrium frequency of *Nod*+ when *Rhiz*+ is initially fixed in the population. In well-mixed environments and in structured environments, rhizopines have no influence of the evolution of cooperation. At intermediate levels of mixing, rhizopines substantially limit nodulation.
(PDF)

Figure S2 **Isoplanes and evolutionary trajectory of approximate linked model.** The blue, green, and yellow represent the zero-growth isoplanes of the *Nod+Rhiz+*, *Nod+Rhiz−*, and *Nod−Rhiz−* genotypes, respectively. The first two isoplanes are overlapping. The red trace represents an evolutionary trajectory. Note the transient increase towards the *Nod+Rhiz+* genotype, and eventual fixation at *Nod+Rhiz−*.
(PDF)

Figure S3 **Gain in frequency of nodulation from the presence of rhizopines.** In lower spatial structure environments, the transient increase in cooperation is more substantial that in highly structured environments. This figure represents the space between the red (*Nod+Rhiz+*) and blue (*Nod+Rhiz−*) curves in the Figure 4a–c. It is interpreted as the increase in frequency of mutualism that would not be realized in the absence of rhizopines.
(PDF)

Text S1 **Costs of nodulation.** A brief discussion of multiple potential costs of nodulation.
(PDF)

Text S2 **Model.** Full exposition of the unlinked and linked models.
(PDF)

Text S3 **Invasion conditions in the linked model.** Identification of the invasion conditions for mutant genotypes in the linked model.
(PDF)

Text S4 **Weak-selection approximation.** Used for visualizing linked model isoplanes.
(PDF)
