# Supplementary material for: Joint Evolution of Kin Recognition and Cooperation in Spatially Structured Rhizobium Populations
Source: PLoS One. 2014 Apr 24;9(4):e95141. doi: 10.1371/journal.pone.0095141 (PMC3999197; doi:10.1371/journal.pone.0095141)
Supplement: Figure S1 — Equilibrium frequency of nodulation is limited by rhizopines. The equilibrium Nod+ frequency is shown as function of spatial structure. The solid curve shows the equilibrium level of cooperation in the absence of rhizopines, while the dashed curve represents the equilibrium frequency of Nod+ when Rhiz+ is initially fixed in the population. In well-mixed environments and in structured environments, rhizopines have no influence of the evolution of cooperation. At intermediate levels of mixing, rhizopines substantially limit nodulation. (PDF) [file pone.0095141.s001.pdf]

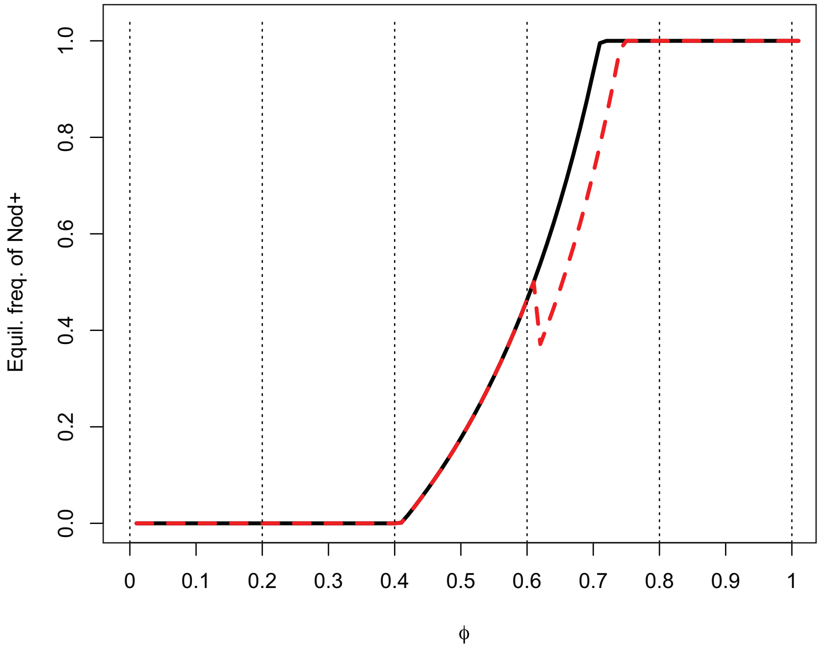

Figure S1. Equilibrium frequency of nodulation is limited by rhizopines. The equilibrium *Nod+* frequency is shown as function of spatial structure. The solid curve shows the equilibrium level of cooperation in the absence of rhizopines, while the dashed curve represents the equilibrium frequency of *Nod+* when *Rhiz+* is initially fixed in the population. In well-mixed environments and in structured environments, rhizopines have no influence of the evolution of cooperation. At intermediate levels of mixing, rhizopines substantially limit nodulation.
