# Supplementary material for: Joint Evolution of Kin Recognition and Cooperation in Spatially Structured Rhizobium Populations
Source: PLoS One. 2014 Apr 24;9(4):e95141. doi: 10.1371/journal.pone.0095141 (PMC3999197; doi:10.1371/journal.pone.0095141)
Supplement: Figure S2 — Isoplanes and evolutionary trajectory of approximate linked model. The blue, green, and yellow represent the zero-growth isoplanes of the Nod+Rhiz+, Nod+Rhiz−, and Nod−Rhiz− genotypes, respectively. The first two isoplanes are overlapping. The red trace represents an evolutionary trajectory. Note the transient increase towards the Nod+Rhiz+ genotype, and eventual fixation at Nod+Rhiz−. (PDF) [file pone.0095141.s002.pdf]

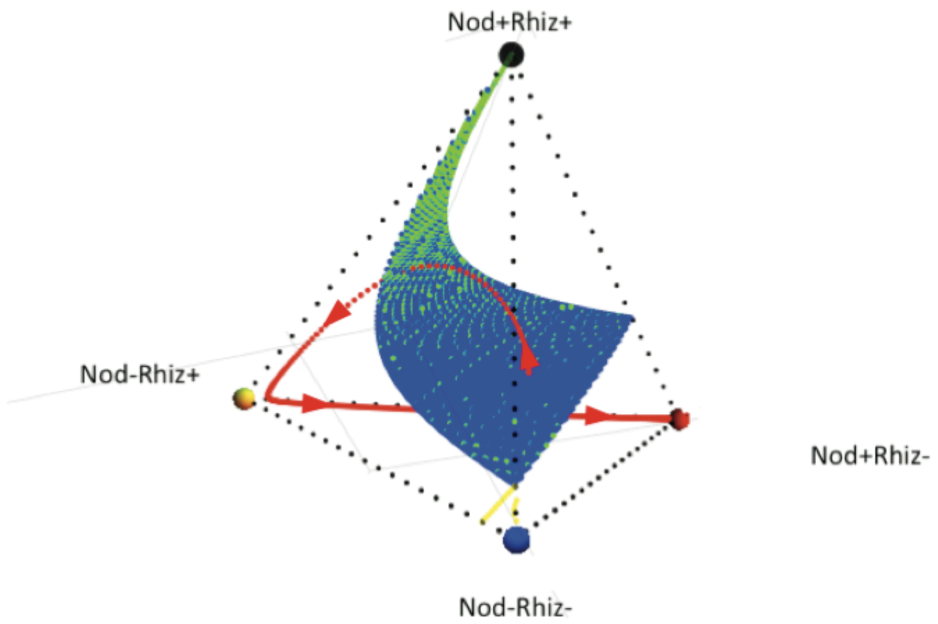

Figure S2. Isoplanes and evolutionary trajectory of approximate linked model. The blue, green, and yellow represent the zero-growth isoplanes of the *Nod+Rhiz+*, *Nod+Rhiz-*, and *Nod-Rhiz-* genotypes, respectively. The first two isoplanes are overlapping. The red trace represents an evolutionary trajectory. Note the transient increase towards the *Nod+Rhiz+* genotype, and eventual fixation at *Nod+Rhiz-*.
