# Supplementary material for: Joint Evolution of Kin Recognition and Cooperation in Spatially Structured Rhizobium Populations
Source: PLoS One. 2014 Apr 24;9(4):e95141. doi: 10.1371/journal.pone.0095141 (PMC3999197; doi:10.1371/journal.pone.0095141)
Supplement: Figure S3 — Gain in frequency of nodulation from the presence of rhizopines. In lower spatial structure environments, the transient increase in cooperation is more substantial that in highly structured environments. This figure represents the space between the red (Nod+Rhiz+) and blue (Nod+Rhiz−) curves in the Figure 4a–c. It is interpreted as the increase in frequency of mutualism that would not be realized in the absence of rhizopines. (PDF) [file pone.0095141.s003.pdf]

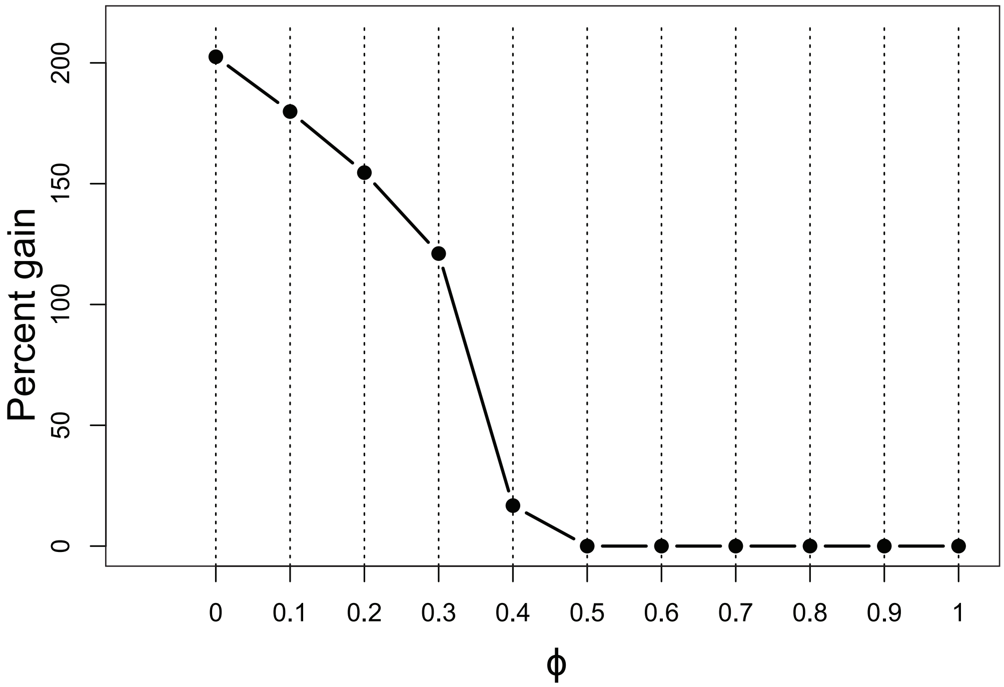

Figure S3. Gain in frequency of nodulation from the presence of rhizopines. In lower spatial structure environments, the transient increase in cooperation is more substantial that in highly structured environments. This figure represents the space between the red (*Nod+Rhiz+*) and blue (*Nod+Rhiz-*) curves in the Figure 4a-c. It is interpreted as the increase in frequency of mutualism that would not be realized in the absence of rhizopines.
