# Supplementary material for: Joint Evolution of Kin Recognition and Cooperation in Spatially Structured Rhizobium Populations
Source: PLoS One. 2014 Apr 24;9(4):e95141. doi: 10.1371/journal.pone.0095141 (PMC3999197; doi:10.1371/journal.pone.0095141)
Supplement: Text S1 — Costs of nodulation. A brief discussion of multiple potential costs of nodulation. (PDF) [file pone.0095141.s004.pdf]

Supporting Information:

Text S1. Costs of nodulation

Joint evolution of kin recognition and cooperation in spatially structured rhizobium populations

Peter C. Zee[1,2*], James D. Bever[1]

[1]Department of Biology, Indiana University, Bloomington, Indiana, United States of America

[2]*current address*: Department of Biology, Stanford University, Stanford, United States of America

Costs of nodulation

The ability to nodulate plants can be a highly beneficial trait in rhizobia for the interspecific mutualism, and for other rhizobia in the rhizosphere. However, the benefit does not come without costs. We identify two potential costs of nodulation. In the main text, we focus on the energetic carriage cost of the symbiotic plasmid where Nod genes are located. The second cost is sterilization of cells as a result of differentiation into bacteroids. Kin selection arguments can be modeled around either of these two costs. Olivieri and Frank [1] modeled the maintenance of the rhizobia-legume mutualism around the sterilization costs of nodulating bacterial cells. This approach has faced criticism in the literature [2], citing the ability of bacteroids to readily de-differentiate into a reproductive state. We do not consider this sterilizing cost in our model because the number of differentiated (or sterilized) individuals in the population is likely a small

proportion of the large bacterial population size. However, we do note that even in the event of de-differentiation, these cells are likely to suffer a decrease in growth rate relative to undifferentiated cells.

References

1. Olivieri I, Frank SA (1994) The evolution of nodulation in rhizobium - altruism in the rhizosphere. J Hered 85: 46-47.

2. West SA, Kiers ET, Simms EL, Denison RF (2002) Sanctions and mutualism stability: why do rhizobia fix nitrogen? Proc Roy Soc Lond B 269: 685-694.
