# Supplementary material for: Joint Evolution of Kin Recognition and Cooperation in Spatially Structured Rhizobium Populations
Source: PLoS One. 2014 Apr 24;9(4):e95141. doi: 10.1371/journal.pone.0095141 (PMC3999197; doi:10.1371/journal.pone.0095141)
Supplement: Text S2 — Model. Full exposition of the unlinked and linked models. (PDF) [file pone.0095141.s005.pdf]

Supporting Information:

Text S2. Model

Joint evolution of kin recognition and cooperation in spatially structured rhizobium populations

Peter C. Zee[1,2*], James D. Bever[1]

[1]Department of Biology, Indiana University, Bloomington, Indiana, United States of America

[2]*current address*: Department of Biology, Stanford University, Stanford, United States of America

Model

In the main text, we present an analysis and interpretation of a two-locus population genetic model investigating the roles of kin recognition and spatial structure on the evolution of cooperation in rhizobium populations. There, we represent the model by the simplified model *Fitness* = $G*E$, where G is a function of constitutive costs and benefits, and E is a function of genotype frequency, environment specific costs and benefits. This cooperative trait (nodulation) is mediated through the bacterium's mutualistic interaction with leguminous plants. Through nodulation, rhizobia provoke the plant to increase carbon rich root exudates, which can be used as a nutrient source for free-living bacterial cells in the rhizosphere. Here, we present the formal model, and analytical solutions for the unlinked model. We also present the full linked model, and solutions based on

approximation of the linked model. Finally, we present analytical expressions describing the conditions necessary for invasion of each genotype in the linked model.

Unlinked Model

The fitness functions of the unlinked model are expressed in terms of the costs and benefits of being in a given nodule environment, weighted by the probability of being found in that environment. In addition, there are also constitutive costs and benefits for some genotypes. For the *Nod* locus, the fitnesses are defined as:

$$w_{Nod+} = (1-c_N)[(1+b_N)(1-c)e(\phi+x(1-\phi))+(1+b_N)f(\phi+x(1-\phi))+y(1-\phi)]$$
$$w_{Nod-} = (1+b_N)(1-c)ex(1-\phi)+(1+b_N)fx(1-\phi)+(\phi+y(1-\phi))$$

For the *Rhiz* locus, the fitnesses are defined as:

$$w_{Rhiz+} = (1-c_r)[(1+b_N)(1-c)x(\phi+e(1-\phi))+(1+b_N)(1-\phi)xf+y(\phi+(1-\phi))]$$
$$w_{Rhiz-} = (1+b_N)(1-c)(1-d)xe(1-\phi)+(1+b_N)x(\phi+f(1-\phi))+y(\phi+(1-\phi))$$

In the main text, these equations are summarized as the product of the function *G* and *E*. In these functions, for each genotype, the terms outside of the square brackets represent *G*, and the terms inside the square brackets are *E*. From these fitness functions, the change in allele frequency for the two loci can be determined:

$$\Delta e = \frac{(1-c_r)(x((1+b_N)c(1-e)\phi-b_Nce+b_N-ce)+1)}{c_r(x(b_N-(c(1+b_N)(1-e)\phi-b_Nce-ce)+1)+(1+b_N)(1-c)d(1-e)x(1-\phi)+cx(1+b_N))-b_Nx}$$

$$\Delta x = \frac{x(1-x)(c_N(b_Nx(1-ce)+cex-1)-\phi(1-c_N(1-x))(ce(1+b_N)-b_N))}{(1-c_Nx)(1-b_Nx(1-ce)-cex)-c_Nx(1-x)\phi(ce(1+b_N)-b_N)}$$

Setting the change in allele frequency to zero, we solve for the equilibrium frequencies of each genotype. In addition to trivial boundary equilibria, we derived the following internal equilibria for *Rhiz* and *Nod*, respectively:

$$\hat{e} = \frac{cx\phi(1+b_N)(1-c_r) - c_r(1+b_N)}{x(1+b_N)(1-\phi)(c(c_r-d)+d)}$$

$$\hat{x} = \frac{\phi(1-c_N)(ce(1+b_N)-b_N) - c_N}{c_N(1-\phi)(ce(1+b_N)-b_N)}$$

Constraint of cooperation by rhizopines

Rhizopines limit the equilibrium frequency of cooperators in the population in the absence of linkage (Figure S1). For each of the initial *Rhiz*+ frequencies investigated, equilibrium cooperator frequency either declined or was unaffected. In no case did rhizopines increase the frequency of mutualism in the population. This represents the two distinct stable equilibria on the phase planes (Figure 3) while the *Nod*+ isocline is moving across the phase plane.

Linked Model

In the linked case, the structure of the fitness functions remains the same. However, because the loci are linked, there are now four genotypes whose frequencies sum to one. These fitnesses are defined as:

$$w_{Nod+Rhiz+} = (1-c_r)(1-c_N)[(1+b_N)(1-c)(\phi+x(1-\phi))+(1+b_N)(y(1-\phi))+(1-x-y)(1-\phi)]$$

$$w_{Nod+Rhiz-} = (1-c_N)[(1+b_N)(1-c)(1-d)(x(1-\phi))+(1+b_N)(\phi+y(1-\phi))+(1-x-y)(1-\phi)]$$

$$w_{Nod-Rhiz+} = (1+b_N)(1-c)(x(1-\phi))+(1+b_N)(y(1-\phi))+(\phi+(1-x-y)(1-\phi))$$

$$w_{Nod-Rhiz-} = (1+b_N)(1-c)(1-d)(x(1-\phi))+(1+b_N)(y(1-\phi))+(\phi+(1-x-y)(1-\phi))$$

As in the unlinked case, the terms outside and inside the square brackets represent the *G* and *E* functions for each genotype. This system of equations does not enable analytical solutions for the equilibria. In order to investigate the equibria in this model, we turn to invasion analyses and an analytical approximation.
