# Supplementary material for: Joint Evolution of Kin Recognition and Cooperation in Spatially Structured Rhizobium Populations
Source: PLoS One. 2014 Apr 24;9(4):e95141. doi: 10.1371/journal.pone.0095141 (PMC3999197; doi:10.1371/journal.pone.0095141)
Supplement: Text S3 — Invasion conditions in the linked model. Identification of the invasion conditions for mutant genotypes in the linked model. (PDF) [file pone.0095141.s006.pdf]

Supporting Information:

Text S3. Invasion conditions in the linked model

Joint evolution of kin recognition and cooperation in spatially structured rhizobium populations

Peter C. Zee[1,2*], James D. Bever[1]

[1]Department of Biology, Indiana University, Bloomington, Indiana, United States of America

[2]*current address*: Department of Biology, Stanford University, Stanford, United States of America

Invasion conditions in the linked model

Because solving the system of equations for the linked model for analytical equilibria is not tenable, we have investigated the conditions for invasion of each genotype by every other genotype. For any parameter combination, this approach allows us to determine the genotypes that are stable.

The invasion conditions for each genotype were determined by assuming fixation of one genotype, and independently comparing fitnesses. This can be visualized by imagining the population at one corner of a simplex, and determining the conditions where it will move along an edge towards another corner. We solve the invasion conditions in terms of ϕ.

Nod+Rhiz+ can be invaded under the following conditions. *Nod+Rhiz-* invades when:

$$\phi < \frac{(1-c)(d-c_r)}{c+d-cd}$$

Nod-Rhiz- invades when:

$$\phi < \frac{(1+b_N)(1-c)(d-c_r-c_N(1-c_r))}{d(1+b_N)(1-c)+b_N-b_Nc-c}$$

and *Nod-Rhiz+* invades when:

$$\phi < \frac{c_N(1+b_N)(1-c)}{b_N(1-c)+c}$$

The *Nod+Rhiz-* genotype can be invaded under the following conditions. *Nod+Rhiz+* invades when:

$$\phi < \frac{-c_r}{c(1-c_r)}$$

Nod-Rhiz- invades when:

$$\phi < c_N\left(\frac{1}{b_N}+1\right)$$

and *Nod-Rhiz+* invades when:

$$\phi < \frac{(1+b_N)(c_N-c_r)}{b_N(1-c_r)}$$

Nod-Rhiz- can be invaded under the following conditions. *Nod+Rhiz+* invades when:

$$\phi < \frac{c_N(1-c_r)-c_r}{(1-c_N)(1-c_r)(b_N(1-c)+c)}$$

Nod+Rhiz- invades when:

$$\phi < \frac{c_N}{b_N - b_N c_N}$$

The solution for the invasion of *Nod-Rhiz-* by *Nod-Rhiz+* is undefined indicating it is not invasible by this genotype. Finally, *Nod-Rhiz+* is invaded under the following conditions.

Nod+Rhiz+ invades when:

$$\phi < \frac{c_N}{(1-c_N)(b_N(1-c)+c)}$$

Nod+Rhiz- invades when:

$$\phi < \frac{c_N - c_r}{b_N - b_N c_N}$$
