# Supplementary material for: Joint Evolution of Kin Recognition and Cooperation in Spatially Structured Rhizobium Populations
Source: PLoS One. 2014 Apr 24;9(4):e95141. doi: 10.1371/journal.pone.0095141 (PMC3999197; doi:10.1371/journal.pone.0095141)
Supplement: Text S4 — Weak-selection approximation. Used for visualizing linked model isoplanes. (PDF) [file pone.0095141.s007.pdf]

Supporting Information:

Text S4. Weak-selection approximation

Joint evolution of kin recognition and cooperation in spatially structured rhizobium populations

Peter C. Zee[1,2*], James D. Bever[1]

[1]Department of Biology, Indiana University, Bloomington, Indiana, United States of America

[2]*current address*: Department of Biology, Stanford University, Stanford, United States of America

Weak-selection approximation

The above invasion analysis does not elucidate the transient dynamics of the system. To gain a qualitative understanding of the dynamics, we have used an approximation that assumes weak selection, thereby eliminating second-order terms. Assuming weak-selection, approximate equilibrium conditions were derived for each of the four genotypes. This approximation leads to simplification because products of selection coefficients (assumed to be small) approach zero, and can be eliminated from the model. We derive expressions for isoplanes through the parameter space, along which genotype frequencies do not change:

$$\hat{x} = \frac{\phi(b_N(c-x)+c)}{(1+b_N)(1-c)d(1-\phi)(1-x+z)-\phi(b_N(1-c)+c)}$$

$$\hat{y} = \frac{(1+b_N)(1-c)dxz(1-\phi) - \phi(1-x)(b_N(1-c)+c)}{(1+b_N)(1-c)dx(1-\phi) - b_N\phi}$$

$$\hat{z} = \frac{\phi(b_N(1-cx) - cx) - (1+b_N)x^2d(1-c)(1-\phi)}{(1+b_N)(1-c)dx(1-\phi)}$$

Using these approximate genotype equilbria, we can plot the isoplanes for each of the four genotypes (Figure S2). Further, we can visualize an evolutionary trajectory in this genotype space simplex (Figure S2; red). Numerical simulations of evolutionary trajectories show a distinct increase in the level of mutualism with increasing rhizopines (i.e., movement towards fixation of the *Nod+Rhiz+* genotype); however, this increase is transient, and eventually the system equilibrates at the *Nod+Rhiz-* genotype.
